# Reactions of Fluoroalkanes with Mg−Mg Bonds: Scope, sp^3^C−F/sp^2^C−F Coupling and Mechanism

**DOI:** 10.1002/chem.201804580

**Published:** 2018-10-05

**Authors:** Greg Coates, Bryan J. Ward, Clare Bakewell, Andrew J. P. White, Mark R. Crimmin

**Affiliations:** ^1^ Department of Chemistry Imperial College London South Kensington London SW7 2AZ UK

**Keywords:** C−F activation, cross-coupling, fluorocarbons, nucleophilic substitution, organomagnesium reagents

## Abstract

sp^3^C−F Bonds of fluoroalkanes (7 examples; 1°, 2° and 3°) undergo addition to a low‐valent Mg−Mg species generating reactive organomagnesium reagents. Further reactions with a series of electrophiles results in a net C−F to C−B, C−Si, C−Sn or C−C bond transformation (11 examples, diversity). The new reactivity has been exploited in an unprecedented one‐pot magnesium‐mediated coupling of sp^3^C−F and sp^2^C−F bonds. Calculations suggest that the sp^3^C−F bond activation step occurs by frontside nucleophilic attack of the Mg−Mg reagent on the fluoroalkane.

The activation and functionalization of sp^3^C−F bonds of fluoroalkanes represents an important and largely unsolved challenge.^1^, ^2^, ^3^ Transformations that use sp^3^C−F bonds as reactive functional groups could potentially open up new avenues in synthesis, including upgrading refrigerants and the late‐stage functionalisation of agrochemicals and pharmaceuticals. Slow progress in this area of research can, in part, be traced to the difficulties associated with the oxidative addition of sp^3^C−F bonds to transition metals. The high sp^3^C−F bond dissociation energy along with the lack of charge stabilisation in the transition state for bond breaking means that defined oxidative addition reactions are incredibly scarce.^4^ In cases where oxidative addition can occur, the resulting metal alkyl complexes are liable to undergo fast β‐hydride elimination. Main group reagents and catalysts offer a complementary approach to transition metal systems. Electrophilic silylium ions,^5^, ^6^ and related species,^7^, ^8^, ^9^ have proven remarkably adept catalysts for fluoride abstraction from fluoroalkanes, while a nucleophilic boryl anion has just emerged as a reagent capable of C−F cleavage of CF_3_H (HFC‐23).^10^ Although we, and others, demonstrated that sp^3^C−F bonds of fluoroalkanes undergo oxidative addition to single‐site Al^I^ complexes,^11^, ^12^, ^13^ no further reactivity of the resultant Group 13 reagents has been reported. In related studies we have shown that the reaction of fluoroarenes with **1 a** occurs by a concerted S_N_Ar‐like addition of the sp^2^C−F bond across the Mg−Mg bond (Scheme 1).^14^, ^15^

**Scheme 1:**
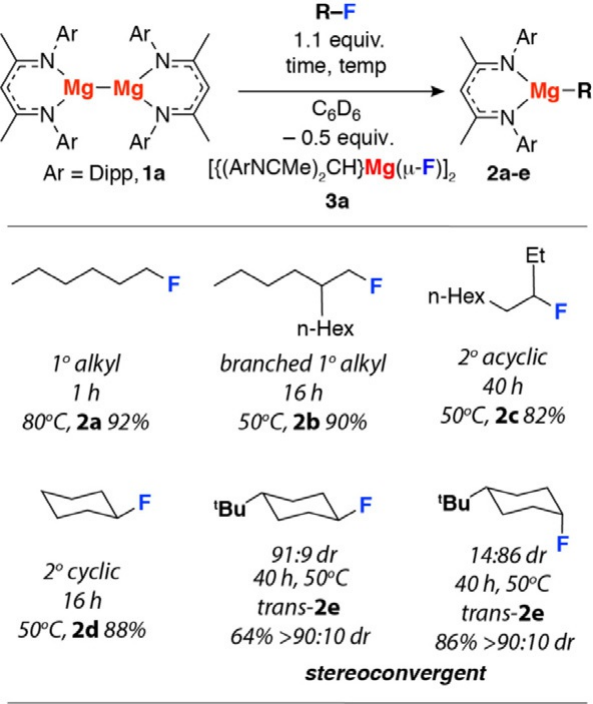
Addition of sp^3^C−F bonds to Mg−Mg bonds. Yields measured by ^1^H NMR spectroscopy by comparison against an internal standard.

Fluorocarbons are often considered inert toward Grignard formation. There is, however, a series of somewhat contradictory reports that metallic magnesium can be used to generate Grignard reagents from fluoroalkanes, provided a suitable initiator (e.g., I_2_, Br_2_, EtBr) is present.^16^, ^17^ Captivated by these studies, we became interested in the reactivity of **1 a** 18, 19, 20, 21, 22, 23 towards fluoroalkanes. Here we show that these reagents activate a variety of sp^3^C−F bonds under mild conditions. The resultant organomagnesium reagents can be used to transfer the alkyl group to boron‐, silicon‐, tin‐ and carbon‐based electrophiles. The latter carbon−carbon bond forming reaction is an unprecedented example of a transition metal free cross‐coupling reaction of two C−F bonds.^24^

Addition of 1.1 equiv of 1‐fluorohexane to a 0.02 m solution of **1 a** in C_6_D_6_ at 80 °C led to the consumption of the Mg‐Mg reagent over 1 h and formation of the magnesium alkyl **2 a** in 92 % yield. **2 a** was characterised by a high‐field triplet resonance in the ^1^H NMR spectrum (*δ*=−0.22 ppm, ^3^ *J*_H–H_=7.9 Hz) assigned to the methylene group adjacent to magnesium and formed alongside the previously characterised magnesium fluoride **3 a**.^25^

The scope of the reaction was considered. A series of substrates was investigated and the organomagnesium complexes **2 b**–**e** were formed in good yields (Scheme 1). The reaction tolerates 1°, 2° and 3° fluoroalkanes along with chain‐branching both adjacent to and remote from the active site. Related organomagnesium complexes crystallise as bridged dimers (1° alkyl) or 3‐coordinate monomers (2°/3° alkyl).^26^, ^27^, ^28^ In the solid‐state **2 a** forms a dimer, bridged by 3‐centre, 2‐electron bonds (Figure 1 a). DFT calculations show that the solid‐state structures likely persist in solution and dimerization of these organomagnesiums only becomes unfavourable with branching of the chain (Figure 1 b). Although **1 a** did not react cleanly with 3° alkyl fluorides, the analogue **1 b** mediates the C−F bond activation of 1‐fluoroadamantane. In this case, the resulting β‐diketiminate stabilised organomagnesium is unstable with respect to Schlenk‐like ligand redistribution preventing its characterisation in solution. Trapping of the organomagnesium with HBpin resulted in direct formation of 1‐adamantylBpin in 69 % yield from **1 b** (Bpin=pinacolatoborane, Figure 1 c).

**Figure 1:**
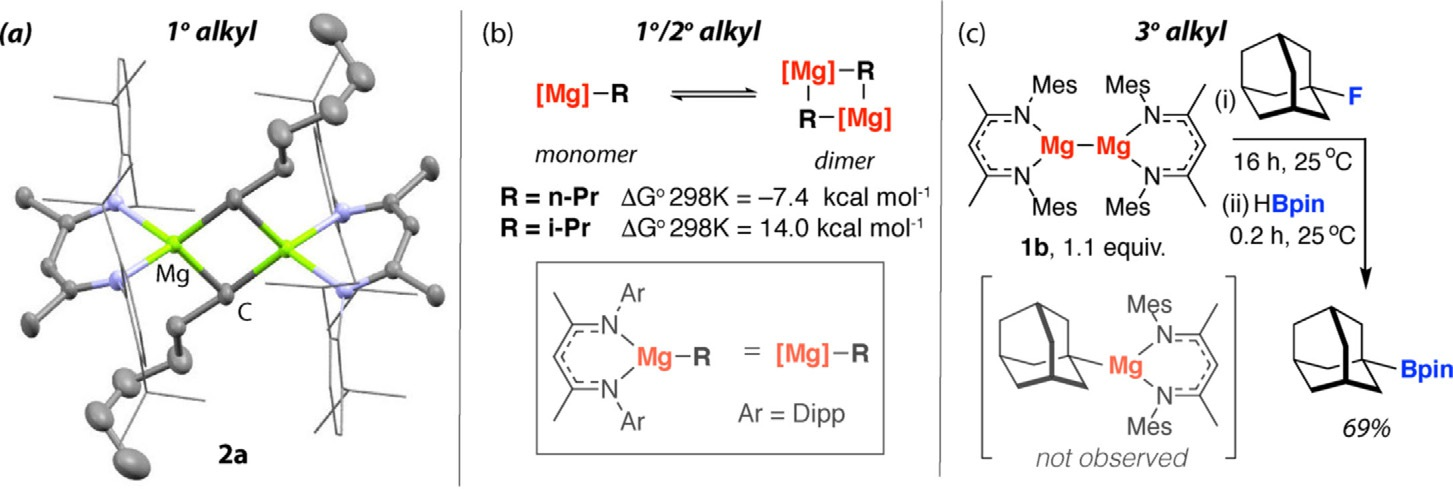
(a) Crystal structure of **2 a**. Selected bond length (Å): **2 a** Mg−C 2.257(3). (b) Calculated Gibbs free energies (kcal mol^−1^) of dimerization of magnesium alkyl complexes. (c) Reaction of **1 b** with 1‐fluoroadamantane and trapping with HBpin.

Initial experiments suggest that, in a case that forms two energetically dissimilar diastereomers, the reaction is stereoconvergent. Hence, *cis* and *trans* 4‐tert‐butylcyclohexyl fluoride both react with **1 a** to give a single diastereomer assigned as *trans*‐**2 e** based on the ^3^ *J*_H‐H_ values of the NMR resonance of the protons adjacent to Mg (Scheme 1). By DFT the *trans* isomer is calculated to be 5.4 kcal mol^−1^ more stable than the *cis* isomer and they likely interconvert by epimerisation of the stereocentre adjacent to magnesium.

Insight into the functional group compatibility of the new transformation was gained by running the reaction of **1 a** with 1‐fluorohexane in the presence of external reagents containing alkenes, alkynes, ethers, 3° amine and pyridine moieties. These additives had little or no impact on the yield of **2 a** (Supporting Information, Scheme S3). In the case of THF and DMAP this experiment led to the formation of the solvates **2 a⋅THF** and **2 a⋅DMAP**, respectively. Substrates including an additional halogen atom on the hydrocarbon chain, such as 1‐iodo‐3‐fluoropropane or 1‐bromo‐5‐fluoropentane, underwent cyclisation to form three‐ or five‐membered hydrocarbon rings (Supporting Information, Scheme S4).^29^

The utility of the new organomagnesium complexes was investigated and specifically the polar Mg^δ+^−C^δ−^ bond derived from sp^3^C−F activation was used as a nucleophilic source of the carbanion. Reaction of mixtures containing **2 a**, formed from C−F activation of 1‐fluorohexane, with HBpin, B_2_pin_2_, B_2_nep_2_, 9‐BBN, H_3_SiPh, HS*n*Bu_3_, or ClS*n*Bu_3_ leads to transfer of the alkyl group from magnesium to the electrophile and results in sp^3^C−B, sp^3^C−Si, and sp^3^C−Sn bond formation, respectively (Bnep=5,5‐dimethyl‐1,3,2‐dioxaborolane, 9‐BBN=9‐borabicyclo[3.3.1]nonane). These reactions are highly efficient, with most proceeding in >80 % yield over the two steps as measured by ^1^H NMR spectroscopy. An exception is the reaction of **2 a** with B_2_nep_2_ which forms *n*‐HexBnep in only 50 % yield (Scheme 2).^30^

**Scheme 2:**
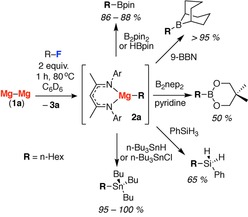
Stepwise sp^3^C−F bond functionalisation resulting in the formation of sp^3^C−B, sp^3^C−Si and sp^3^C−Sn bonds. For full details of these experiments see the supporting information.

Encouraged by the ease of nucleophilic addition to main group electrophiles, we turned our attention to intermolecular carbon−carbon bond formation by the heterocoupling of two C−F bonds. **2 a**, generated directly from 1‐fluorohexane, adds to perfluoroarenes under forcing conditions (Scheme 3). The reaction of in situ generated **2 a** with hexafluorobenzene forms **4 a** as evidenced by the emergence of a new triplet resonance in the ^1^H NMR spectrum (*δ*=2.29 ppm, ^3^ *J*_H–H_=7.7 Hz) assigned to the methylene protons adjacent to the aromatic ring. The scope of this reaction was expanded and the overall yields of cross‐coupled products **4 a**–**e** while modest, 34–72 %, represent a combination of two steps and an average 60–85 % yield for each C−F bond cleavage reaction. Although related reactions of organomagnesium reagents with perfluoroarenes are known,^31^, ^32^, ^33^ this represents the first transition metal free procedure for C−C bond formation by the coupling of two C−F bonds.

**Scheme 3:**
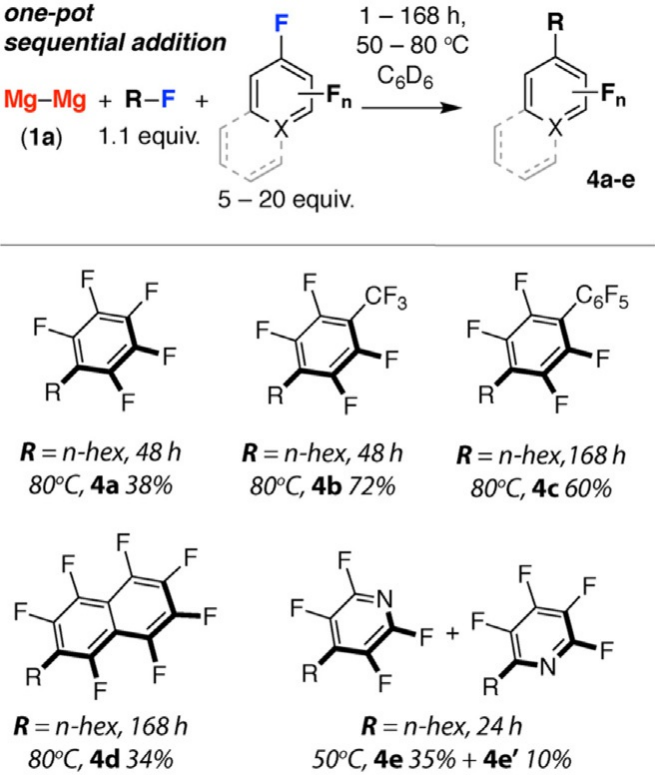
Carbon−carbon bond formation by double carbon−fluorine bond activation. Yields measured by ^1^H NMR by comparison against an internal standard.

To gain a deeper understanding of the C−F bond cleavage steps involved in the carbon−carbon bond forming sequence, a series of calculations were undertaken on the reaction of 1‐fluoropropane^34^ with hexafluorobenzene using the B3PW91 functional and a hybrid basis set (Figure 2 a). We have previously benchmarked the computational methods used herein against experimentally determined activation parameters.^15^

**Figure 2:**
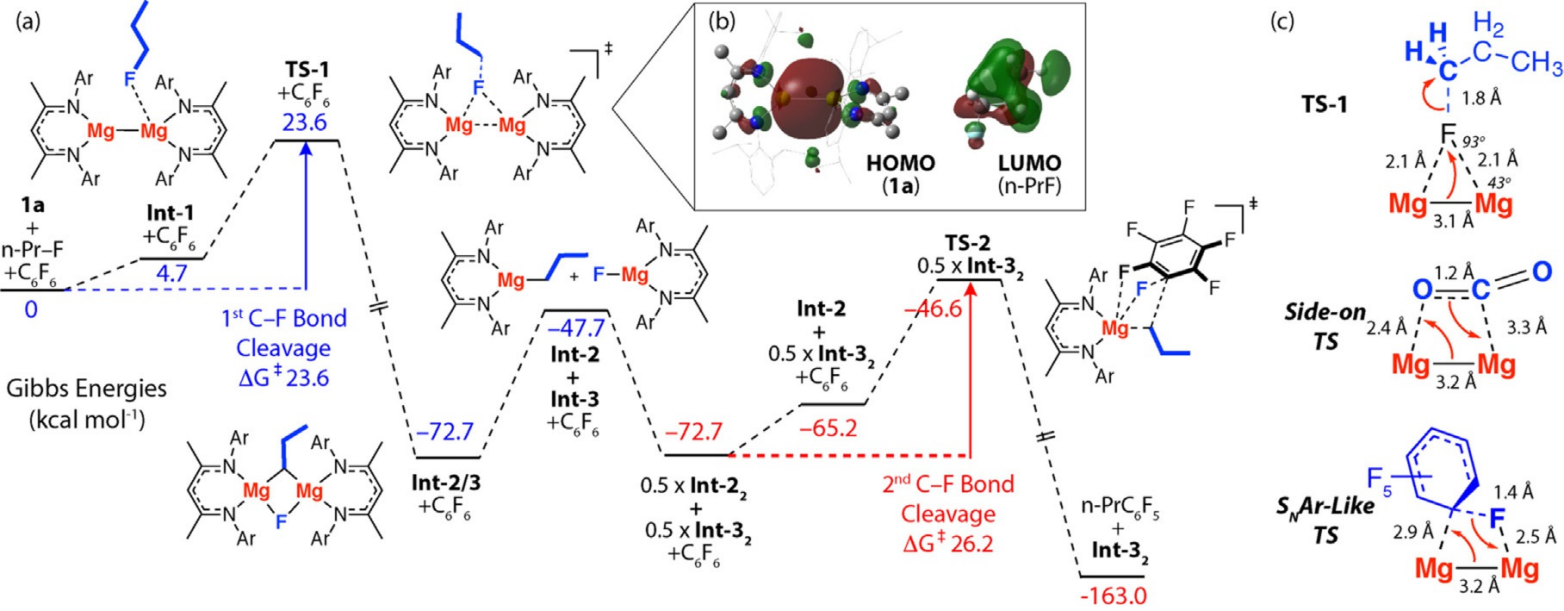
(a) Calculated potential energy surface for the sequential reaction of **1 a** with *n*‐Pr−F and C_6_F_6_. Gibbs energies in kcal mol^−1^. (b) HOMO and LUMO of **1 a** and 1‐fluoropropane, respectively. (c) Geometry of **TS‐1** and comparison against related TS.

The initial endergonic coordination of 1‐fluoropropane at **1 a** to form **Int‐1**, is followed by C−F bond cleavage in **TS‐1** ultimately leading to the formation of **Int‐2/3**.^35^ Schlenk‐like redistribution of two equivalents of **Int‐2/3** forms the experimentally observed products **Int‐2_2_** and **Int‐3_2_**. While the dissociation of **Int‐2/3** into the monomeric fragments **Int‐2** and **Int‐3** required for redistribution is endergonic Δ*G* ^o^_298 K_=25.3 kcal mol^−1^, this energy barrier represents complete dissociation and, as such, is an upper limit of the activation energy. Overall this Schlenk‐like redistribution is thermoneutral. The second C−F bond cleavage step forms the carbon−carbon bond and proceeds by nucleophilic addition of the newly formed magnesium alkyl complex to the electron‐deficient arene. Dissociation of **Int‐2_2_** is required to access the reactive three‐coordinate magnesium alkyl species **Int‐2**, and is on the way to the concerted S_N_Ar transition state **TS‐2**.^36^, ^37^ In combination these steps lead to a high activation barrier for carbon−carbon bond formation, Δ*G* ^≠^_298 K_=26.2 kcal mol^−1^.^38^

The unusual geometry of **TS‐1** warrants further discussion. **TS‐1** contains a near planar arrangement of Mg, C and F atoms in which the C−F bond orientates itself perpendicular to the Mg−Mg bond with the fluorine atom approaching head‐on. The C−F bond stretches to 1.84 Å from 1.39 Å in 1‐fluoropropane, the Mg−F distances are short (≈2.1 Å) while both Mg–C distances are long (>3.6 Å). A similar transition state was located for the reaction of **1 a** with 2‐fluoropropane. **TS‐1** bears all the hallmarks of front‐side nucleophilic attack in an S_N_2 mechanism; the carbon substituent takes the role of the leaving group and the electron‐pair between the magnesium atoms of **1 a** the role of the nucleophile.^39^, ^40^, ^41^, ^42^ This geometry is starkly different to that observed in the side‐on and S_N_Ar like transition states calculated for the reaction of **1 a** with CO_2_ and C_6_F_6_, respectively.^15^, ^43^ While all these processes can be classified as oxidative additions from the perspective of the main group reagent there are significant deviations in the TS geometries (Figure 2 c).

Frontside nucleophilic attack, taught as an unfavourable pathway to undergraduate students, has been modelled in dynamics calculations on nucleophilic substitution reactions of alkyl halides.^39^, ^40^, ^41^ These pathways have been shown, universally, to be prohibitively high in energy when compared to back‐side nucleophilic attack. In the current case, it appears the unusual nature of **1 a** overrides the standard selectivity. There is limited precedent for the geometry of **TS‐1**. Eisenstein and co‐workers have postulated that a cerocene hydride attacks C_6_F_6_ through a transition state involving an end‐on H–F−C interaction.^44^

The Mg−Mg reagent **1 a** possesses a non‐nuclear local maximum in electron density at the centre of the metal−metal bond that acts as a highly nucleophilic electron‐pair.^45^, ^46^ Second‐order perturbation calculations on **TS‐1** show donor‐acceptor interactions from not only the Mg−Mg σ‐bond to the low‐lying σ*(F–C) orbital of the fluoroalkane (37 kcal mol^−1^) but also from the filled F π‐orbitals to the empty σ*(Mg–Mg) orbital (7 kcal mol^−1^). This latter interaction contributes to the stabilisation of the frontside TS as the electrostatic interactions between fluorine and magnesium atoms anchor the C−F bond in place and polarise it. In **TS‐1**, the hydrocarbon chain acts as a leaving group. This moiety adopts carbanion character and following breaking of the C−F bond migrates directly to magnesium (Supporting Information, movie). The carbanion character is evidenced by the NPA charges on the carbon atom in **TS‐1** which is more negative than that in **Int‐1** alongside the deviation of the carbon centre from sp^3^ to sp^2^ hybridised (degree of pyramidalization; **Int‐1**=42 %, **TS‐1**=12.5 %).^47^

In summary, we report a new reaction that transforms sp^3^C−F bonds into reactive sp^3^C−Mg bonds. This methodology can be considered as an equivalent of Grignard formation that occurs in homogeneous solution and allows expansion of the substrate scope to include fluorocarbons. The organomagnesium products react with a series of electrophiles leading to the development of an unprecedented carbon−carbon bond forming reaction that couples two C−F bonds. A preliminary assessment of the mechanism hints that sp^3^C−F bond activation occurs by a remarkable pathway involving frontside nucleophilic attack. We are currently investigating the stereospecifity of the reaction of **1 a** (and related reagents) with fluoroalkanes alongside a more detailed study of the stereointegrity of the resulting organometallics.

## Conflict of interest

The authors declare no conflict of interest.

## Supporting information

As a service to our authors and readers, this journal provides supporting information supplied by the authors. Such materials are peer reviewed and may be re‐organized for online delivery, but are not copy‐edited or typeset. Technical support issues arising from supporting information (other than missing files) should be addressed to the authors.

Supplementary

Supplementary
