# Supplementary material for: Reactions of Fluoroalkanes with Mg−Mg Bonds: Scope, sp3C−F/sp2C−F Coupling and Mechanism
Source: Chemistry. 2018 Oct 5;24(61):16282–6. doi: 10.1002/chem.201804580 (PMC6471154; doi:10.1002/chem.201804580)
Supplement: Supplementary file 2 — Supplementary [file CHEM-24-16282-s002.pdf]

CHEMISTRY
A European Journal

Supporting Information

Reactions of Fluoroalkanes with Mg—Mg Bonds: Scope, sp^3C—F/sp^2C—F Coupling and Mechanism

Greg Coates, Bryan J. Ward, Clare Bakewell, Andrew J. P. White, and Mark R. Crimmin*[a]

chem_201804580_sm_miscellaneous_information.mp4
